# Chronic Skin Lesions as the Presentation of Diffuse Cutaneous Leishmaniasis in the HIV-Infected Woman: A Case Report and Review of Literatures

**DOI:** 10.31661/gmj.v8i0.1294

**Published:** 2019-01-01

**Authors:** Mohammad Ali Davarpanah, Amirreza Dehghanian, Ali Akbari, Behnam Dalfardi

**Affiliations:** ^1^Department of Internal Medicine, Shiraz University of Medical Sciences, Shiraz, Iran; ^2^Trauma Research Center, Shiraz University of Medical Sciences, Shiraz, Iran; ^3^Department Of Pathology, Shiraz University of Medical Sciences, Shiraz, Iran; ^4^Department of Anesthesiology, Shiraz University of Medical Sciences, Shiraz, Iran; ^5^Student Research Committee, Shiraz University of Medical Sciences, Shiraz, Iran

**Keywords:** HIV, Infectious Disease Medicine, Leishmaniasis, Skin Diseases

## Abstract

**Background:**

Cutaneous leishmaniasis (CL) is the most form of leishmaniasis that caused by intracellular parasites, *Leishmania*.

**Case Report:**

A 39-year-old woman, known case of HIV infection, presented with a 6-month history of skin lesions initially on her face, then extending onto the chest, abdomen, and extremities. Laboratory examinations revealed leukopenia and a CD4 cell count of 280 cells / mm3. A biopsy was taken from skin lesions, and histopathological studies showed aggregates of macrophages filled with numerous Leishman bodies, the diagnosis of diffuse CL was confirmed. Consequently, she received liposomal amphotericin B (total dose of 40 mg/kg) as a case of diffuse CL. The skin lesions showed significant improvement after completion of treatment.

**Conclusion:**

Diffuse CL should be considered as a differential diagnosis in all patients with diffuse skin lesions mainly in the cases that suffer from disorders of cell-mediated immunity.

## Introduction

Leishmaniasis is a disease caused by intracellular flagellated protozoans belonging to the genus *Leishmania* [1]. It is a vector-borne infection, and the bite of infected female phlebotomine sandflies is responsible for its transmission [2]. These parasites can cause different disease forms in human, including cutaneous, mucocutaneous, and visceral involvement [1].Cutaneous leishmaniasis (CL) is the most common form of leishmaniasis that caused by various species of *Leishmania* parasites [1]. Also, its annual incident was reported at approximately 0.7 to 1.3 million cases [3]. Nowadays, CL is known as a public health problem that affects more than 60 countries around the world, particularly those located in tropical and subtropical regions [3]. According to the available epidemiologic data, Brazil, Afghanistan, Syria, Iran, Pakistan, Algeria, Peru, Colombia, and Saudi Arabia have the most prevalence of CL [4]. Clinical manifestations of CL are usually limited to the skin, and affected patients do not experience any other symptoms [3].

## Case Presentation

**Figure 1:**
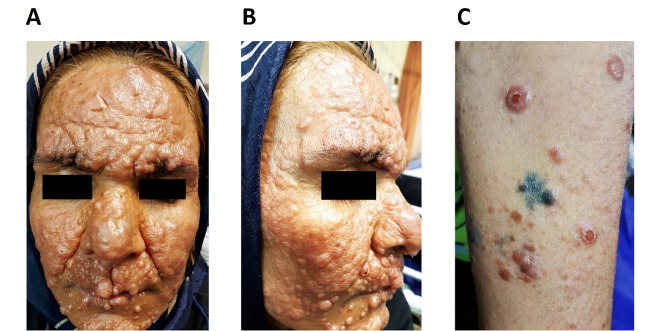
Skin lesions of the face (A and B), and the upper extremities (C).

**Figure 2:**
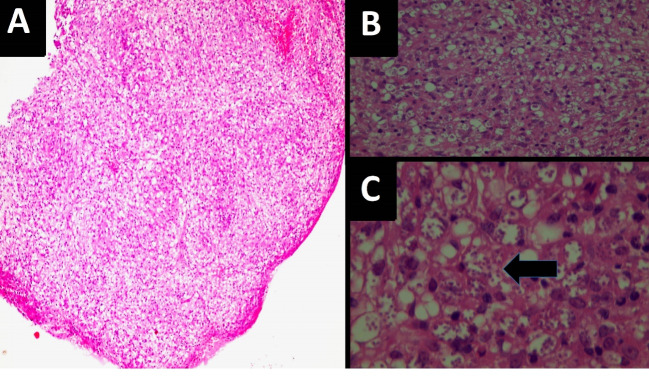
Microscopic images of the skin lesions. **A:** Infiltration of inflammatory cells such as histiocytes-macrophages, lymphocytes, and plasma cells in the dermis. Epidermis shows thinning without ulceration (Hematoxylin and Eosin, ×100). **B** and **C:** Aggregates of macrophages filled with numerous leishman bodies (arrow) are shown in the high power field images (Hematoxylin and Eosin, ×200 and ×400, respectively).

A 39-year-old woman ‒ a known case of HIV infection from 1 year ago ‒ who was admitted to our hospital (Shahid Faghihi Hospital, Shiraz, Iran) with a 6-month history of skin lesions that initially was present on her face, later extending onto the chest, abdomen, and extremities. Our physical examination revealed varying size skin papules and nodules (Figure-1). Laboratory data showed the only leukopenia (WBC: 1700 cells / mm^3^) and a CD4 cell count of 280 cells / mm^3^. Skin lesion biopsy was performed, and histopathological studies showed a busy dermis with infiltration of many inflammatory cells, including histiocytes-macrophages, lymphocytes, and plasma cells (Figure-2). High power field images revealed aggregates of macrophages filled with numerous leishman bodies (Figure-2). These findings confirmed the diagnosis of CL. Regarding patient’s immune deficiency and the extent of skin lesions, the diagnosis of diffuse CL was made. She received liposomal amphotericin B with a total dose of 40 mg/kg as a case of diffuse CL. Her skin lesions showed significant improvement in follow-up visits after completion of treatment (particularly after six months).

## Discussion

Diffuse CL is a rare condition occurring mainly in cases with a defect of cell-mediated immunity, like those suffering from acquired immune deficiency syndrome (AIDS). It is suggested that in such patients, dissemination of the *Leishmania* amastigotes through the infected macrophages in different areas of the skin are responsible for diffuse CL [5]. However; the precise immune-pathogenic mechanisms contributing to the development of this condition remained unclear [6]. A single and painless nodule could be the initial presentation of diffuse CL. However; after a while, as the disease progresses, patients will experience multiple erythematous, flesh-colored papulonodular cutaneous lesions, infiltrative plaques, or macules [2]. These lesions have a high parasite load and may involve skin in different body regions, including the face, upper and lower limbs, etc. [2]. However, visceral involvement is not seen in these patients [7]. The treatment of diffuse CL could be difficult, and relapse is frequent [8]. There are few reports of diffuse CL from Iran. In 2018, Hashemi *et al*. [9] reported a 78-year-old man with multiple papules, crusted and severely ulcerated cutaneous lesions that involved his arms and chest. This patient was known the case of chronic obstructive pulmonary disease and had a history of opium abuse. Their workups including direct smear and a punch biopsy of skin lesions, as the well as molecular study, indicated the diagnosis of *L*. *major* infection. Alborzi *et al*. [10] in 2006, described a 15-year-old girl who had a complaint of papular skin lesions, firstly appeared on her left forearm, next progressed to numerous, small-size papulonodular and painless lesions on her face, back, and extremities. Their examinations resulted in the diagnosis of visceral leishmaniasis and disseminated cutaneous leishmaniasis caused by *L. tropica*.

## Conclusion

Although diffuse CL is a rare condition, it should be considered in cases with diffuse skin lesions particularly in the patients that suffer from disorders of cell-mediated immunity. Of note, epidemiological data on infectious diseases, especially *Leishmania* infection can be a helpful guide regarding this disease.

## Conflict of Interest

None declared.
